# Event and Comment

**Published:** 1923-01

**Authors:** 


### DENTAL REGISTER

THE MAGAZINE OF DENTISTRY

Vol. LXXVII JANUARY, 1922 No. 1

### EVENT AND COMMENT

One of the subjects on which the eminent physician from France, M. Coue, has not given us any enlightenment is whether this celebrated formula will disspell the toothache. Is it possible by autosuggestion and repeating twenty times the littany "Every day—in every way—I am getting better and better," to cure dental ills? An abscessed tooth with the resultant pain would be a remarkable test of Coue's autosuggestion. If the Coue process is a success, there is opportunity for specialists in autosuggestion anesthetics for alleviation of operative pain. It is possible no doubt that the application of some of Coue's principles by the patient himself or herself would make the patient less apprehensive of pain but whether such disregard of pain can cure the cause of the pain, such as an abscessed tooth, is another question. Can autosuggestions tighten loose teeth, is another interesting speculation. Coue may have in his principles and clever verbal formula the secret to the cure of pyorrhea, at least a patient might be led to try autosuggestion where instrumentation failed.

Another interesting contemporary principle of healing is advanced by Dr. Albert Abrams of California. The electric reactions of Abrams are vibrations set up by electric tuning in on the particular vibrations of the diseased tissue itself. The theory is that human tissue has a vibratory frequency in health and deviation from the normal frequency in disease or ill health. An electrical device that catches up the wave length and tunes in on the frequency of the diseased tissue vibration is an invention of Dr. Abrams. The bacteria are vanquished in a storm of electric reaction vibrations which correspond to their own vibration. Of course the scientific elucidation of the electric principles involved in this discovery are far beyond the lay mind and beyond a good many other minds besides. However, as a laboratory principle, it is held that a vibration of the air or ether, if the same as the vibration of an object, such as a glass, which has been struck with a force will shatter the glass to bits. The problem is to find the particular vibratory frequencies of various diseased parts and with the electric reaction outfit set up corresponding and equivalent electric vibrations. These will penetrate and heal the tissue which is diseased. This may be an explanation why radium is beneficial and healing in cancer because although not controllably variable in its vibratory value it has a natural vibratory force which in many cases exactly corresponds with the vibration of the molecules of diseased parts.

Both of these discoveries are worthy of notice and while it may take more wisdom than we possess at the present time to understand them, we must remember that there are untold yet many things which the universe will eventually know. Among them these problems of cure of physical ailments of which the human mind catches glimpses of the ultimate healing methods.—*Votre Ami Dentaire.*

---

The Research Division of the Dentists Supply Company of New York announces a course in denture construction in Cincinnati by Dr. Turner, of the Research Division. The date set for this course is March 19 to 24th, 1923. Registration for this course will be received direct or by local dental supply houses.

Mr. C. E. Perkins is secretary of the Research Division of the Dentists Supply Company and is supplying application cards to be used in securing registrations for this course. More detail notice of this splendid denture course will be given in the next issue of the Dental Register.

